# The complement system in Alzheimer’s disease: mechanisms, biomarkers, and therapeutic implications

**DOI:** 10.3389/fimmu.2026.1798288

**Published:** 2026-04-15

**Authors:** Guokang Liu, Boshao Deng, Xiao Han, Yunpei Zhao, Shiyu Zeng, Suqin Yu, Lulu Wang, Siyi Wang, Yufei Wu, Linyuan Cai, Yuzhang Wu, Hong Yang, Jian Chen

**Affiliations:** 1 Department of Immunology, Army Medical University (Third Military Medical University), Chongqing, China; 2 Department of Pharmacy, Southwest Hospital, Third Military Medical University, Chongqing, China; 3 State Key Laboratory of Oral Diseases & National Center for Stomatology & National Clinical Research Center for Oral Diseases, Department of Prosthodontics, West China Hospital of Stomatology, Sichuan University, Chengdu, Sichuan, China; 4 Anyue County People’s Hospital, Ziyang, Sichuan, China

**Keywords:** Alzheimer’s disease, biomarkers, complement system, disease-modifying therapy, neuroinflammation, synaptic pruning

## Abstract

Alzheimer’s disease (AD) is a multifactorial neurodegenerative disorder in which synaptic loss is closely associated with cognitive decline. Although the amyloid hypothesis has long dominated AD research, the limited efficacy of amyloid-targeted therapies highlights the need to explore additional pathogenic mechanisms. Increasing evidence indicates that dysregulation of the complement system plays a critical role in AD, linking genetic risk, protein aggregation, neuroinflammation, and neurodegeneration. Under physiological conditions, complement signaling is essential for neural development and synaptic refinement; however, in AD, aberrant activation contributes to excessive synaptic pruning and sustained inflammatory responses. As a result, complement components have attracted attention as potential biomarkers and therapeutic targets, despite limitations in disease specificity. This review summarizes current advances in understanding complement system alterations in AD, discusses their roles in disease pathogenesis, and highlights emerging complement-targeted therapeutic strategies, as well as remaining challenges related to intervention timing, patient stratification, and blood–brain barrier delivery.

## 1 Introduction

Alzheimer’s disease (AD) is the most common cause of dementia, accounting for an estimated 60%–80% of all cases. In the United States alone, approximately 7.2 million individuals aged 65 years and older are living with Alzheimer’s dementia in 2025. This number is projected to rise substantially, reaching nearly 12.7 million by 2050 and 13.8 million by 2060, largely driven by population aging (1).

The amyloid cascade hypothesis has long held a central position in the field of Alzheimer’s disease(AD) research. Under this conceptual framework, the pathological accumulation of Aβ peptides is viewed as the primary driver of disease pathogenesis, precipitating a sequence of downstream events that includes tau hyperphosphorylation, synaptic failure, and eventual neuronal death (2). Yet, the validity of this model has faced increasing scrutiny in recent years. While treatment with anti-Aβ monoclonal antibodies—notably lecanemab and donanemab—has proven effective at reducing cerebral amyloid burdens in early-stage patients, the clinical benefits observed to date have been relatively modest (3, 4). Such outcomes imply that therapeutic strategies targeting amyloid pathology in isolation may not be adequate to fundamentally alter the course of the disease.

As a result, the field has progressively shifted toward a more integrative, multifactorial “network disease” framework for AD. Within this broader conceptual model, complement-mediated neuroinflammation has gained recognition as a key pathological axis that intersects genetic risk, protein aggregation, vascular and metabolic stress, and neurodegenerative processes (5). Consistent with this view, genetic studies have identified multiple complement-related loci—including CR1, clustern(CLU), C1q, and TREM2—as significant contributors to AD susceptibility (6).

Neuropathological investigations further reveal pronounced activation of the classical complement cascade (C1q–C3–MAC) in the vicinity of Aβ plaques and tau tangles, a process closely associated with aberrant synaptic pruning and microglia-mediated neurotoxicity (7–9). In addition, sustained complement activation can engage self-reinforcing pathological loops involving vascular dysfunction, oxidative stress, and mitochondrial impairment, which together converge on progressive synaptic loss and cognitive decline (10).

An integrative perspective, which considers complement system dysregulation as a core pathogenic driver, offers a valuable framework for interpreting the considerable clinical heterogeneity in AD and the variable efficacy of current therapeutics. This view aligns well with the recognized temporal trajectory of AD, which evolves from preclinical biomarker changes to mild cognitive impairment and, ultimately, to established dementia (11). The ongoing development of multi-omics platforms, refined neuroimaging techniques, and sensitive fluid biomarker assays is progressively clarifying the spatiotemporal dynamics of complement pathway engagement throughout the disease continuum (12). The convergence of evidence from these methodologies positions complement activation as an early event in the disease cascade, thereby strengthening the rationale for its investigation as a therapeutic target, distinct from a mere secondary outcome of neuronal damage.

This review synthesizes current knowledge regarding the role of the complement system in the complex pathogenesis of AD. We will place special focus on the crosstalk between complement and key AD elements, including genetic risk factors, the aggregation of misfolded proteins, microglial immune activity, vascular and metabolic dysregulation, and emerging environmental risk factors (13). Moving beyond a model of additive contributions, we will explore how complement signaling both influences and is shaped by disease stage and progression, ultimately defining evolving pathogenic hierarchies over time. By integrating foundational mechanistic understanding with recent experimental and clinical findings, we seek to clarify the translational relevance of complement pathways for biomarker development, targeted therapeutic strategies, and refined patient stratification. Our objective is to thereby inform the evolution of more precise, stage-adapted paradigms for AD management.

## 2 Complement system in central nervous system and neurodegenerative diseases

**Figure 1:**
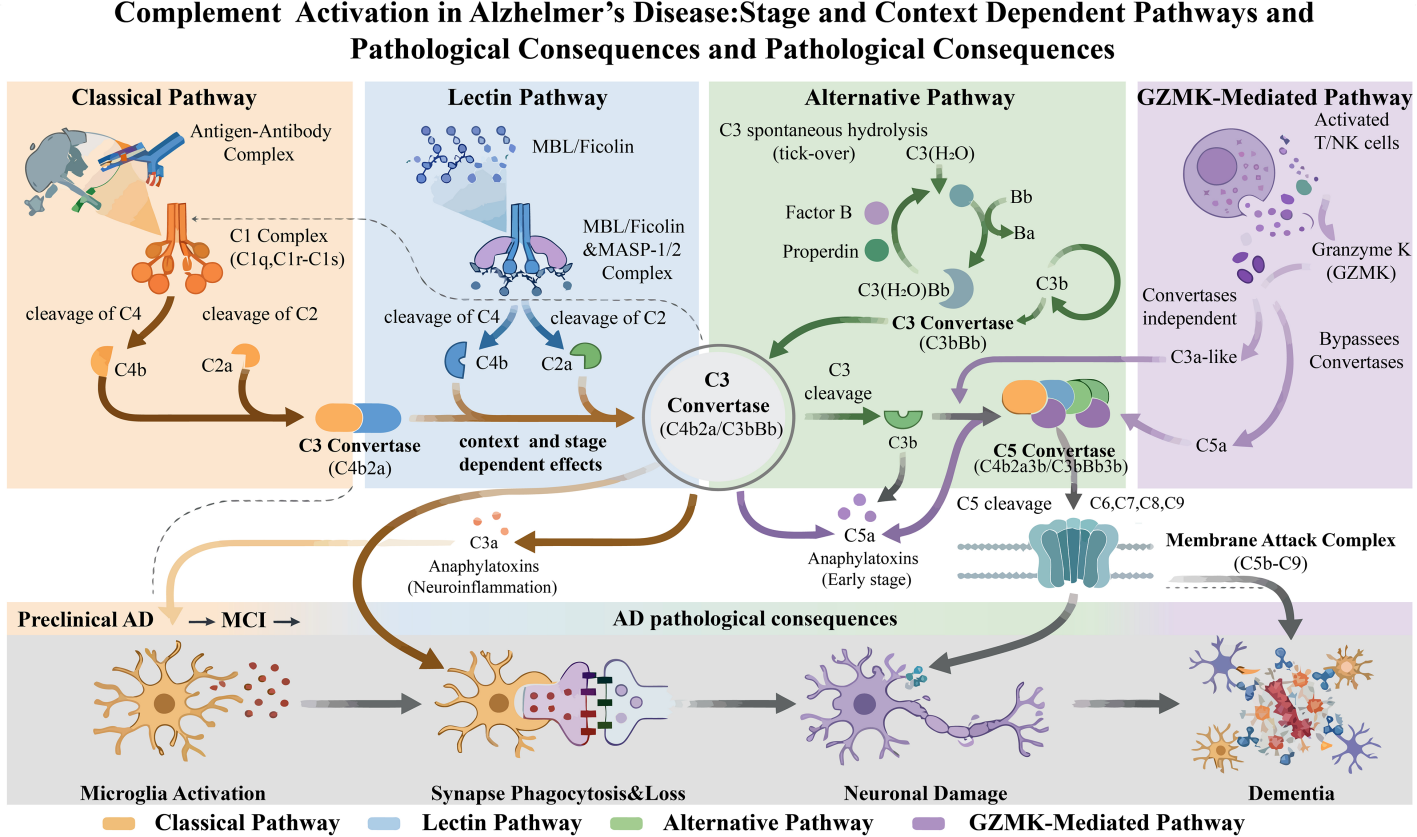
Overview of the complement system activation pathways. This diagram illustrates the three classical complement activation pathways and an additional newly described, independent complement activation pathway.

The complement system is a complex network of soluble and membrane-bound proteins (14). Comprising over 50 soluble and membrane-bound components, regulators, and receptors, it typically circulates in an inactive precursor state, ready to be cleaved into smaller, activated fragments when necessary (15). In the central nervous system (CNS), the complement system functions as a “double-edged sword”: under normal physiological conditions, it orchestrates neural development, synaptic pruning, and homeostatic maintenance; however, when pathologically over-activated, it can drive synapse loss, neuroinflammation, and neurodegeneration (16). Most complement proteins are secreted by glial cells in a healthy state, with the enzymatic components constitutively present in blood, but they require activation to propagate the cascade. Traditionally, the complement system is thought to have three activation pathways: the classical, alternative, and lectin pathways (17). Recent evidence, however, suggests the existence of additional tissue-specific activation mechanisms. Notably, granzyme K (GZMK) has been reported to initiate a non-canonical mode of complement activation independent of the traditional pathways (**Figure 1**) (18). Interestingly, prior clinical studies have identified altered GZMK expression in patients with Alzheimer’s disease, although its role in complement activation was not recognized at the time. This emerging link raises the possibility that non-canonical complement activation mechanisms may contribute to early inflammatory dysregulation in AD (19).

Although complement components were traditionally considered liver-derived and largely excluded from the central nervous system (CNS) by the blood–brain barrier, it is now well established that CNS-resident cells locally synthesize complement proteins and express their cognate receptors (20). Microglia constitute the principal source of C1q within both the healthy and diseased brain, whereas astrocytes contribute substantially to the production of C3 and additional complement components (21, 22). Neurons, beyond serving as targets of complement-mediated remodeling, express complement receptors such as CR3 and C3aR, enabling them to directly sense complement activation (23). This intrinsic capacity for local complement synthesis underscores the functional autonomy of CNS complement regulation, independent of peripheral hepatic production (**Table 1**).

**Table 1:** Cellular sources of complement proteins in the brain. This table illustrates the sources of complement proteins, complement receptors, and complement regulatory factors in the central nervous system.

Cell type	Complement protein	Complement receptor	Complement regulators
Microglia	C1q (24), C3 (25)	CR1 (26), CR3 (27)	SIRPa (28)
	C4 (25), C5 (29)	C3aR (30) C5aR (30)	CD59 (31), FH (32)
	C9 (33)	C5aR2 (29)	FI (34)
Astrocytes	C1q (35), C1r (33) C1s (33), C2 (33) C3 (35), C4 (35) C6 (33)	CR1 (36)	CD59 (37), CLU (29) FH (32), FI (38) C1INH (38) GLU (39) CD55 (40), C4BP (41)
Neurons	C1q (42), C5 (43)	C3aR (44) C5aR1 (45)	CD46 (46) CD59 (47) CLU (48)
Oligodendrocytes	C4 (49)		CD46 (47), CD59 (50)
Endothelial cells		C3aR (51) C5aR1 (52)	CD46 (47), CD59 (50), Cfh (25)

Complement signaling within the CNS is therefore highly compartmentalized. Rather than functioning as a diffuse systemic cascade, complement activation is spatially restricted to defined cellular and synaptic domains (53). Such precision allows complement to support circuit refinement under physiological conditions, yet also renders selectively vulnerable neural networks particularly susceptible to dysregulated amplification once regulatory thresholds are exceeded.

**Figure 2:**
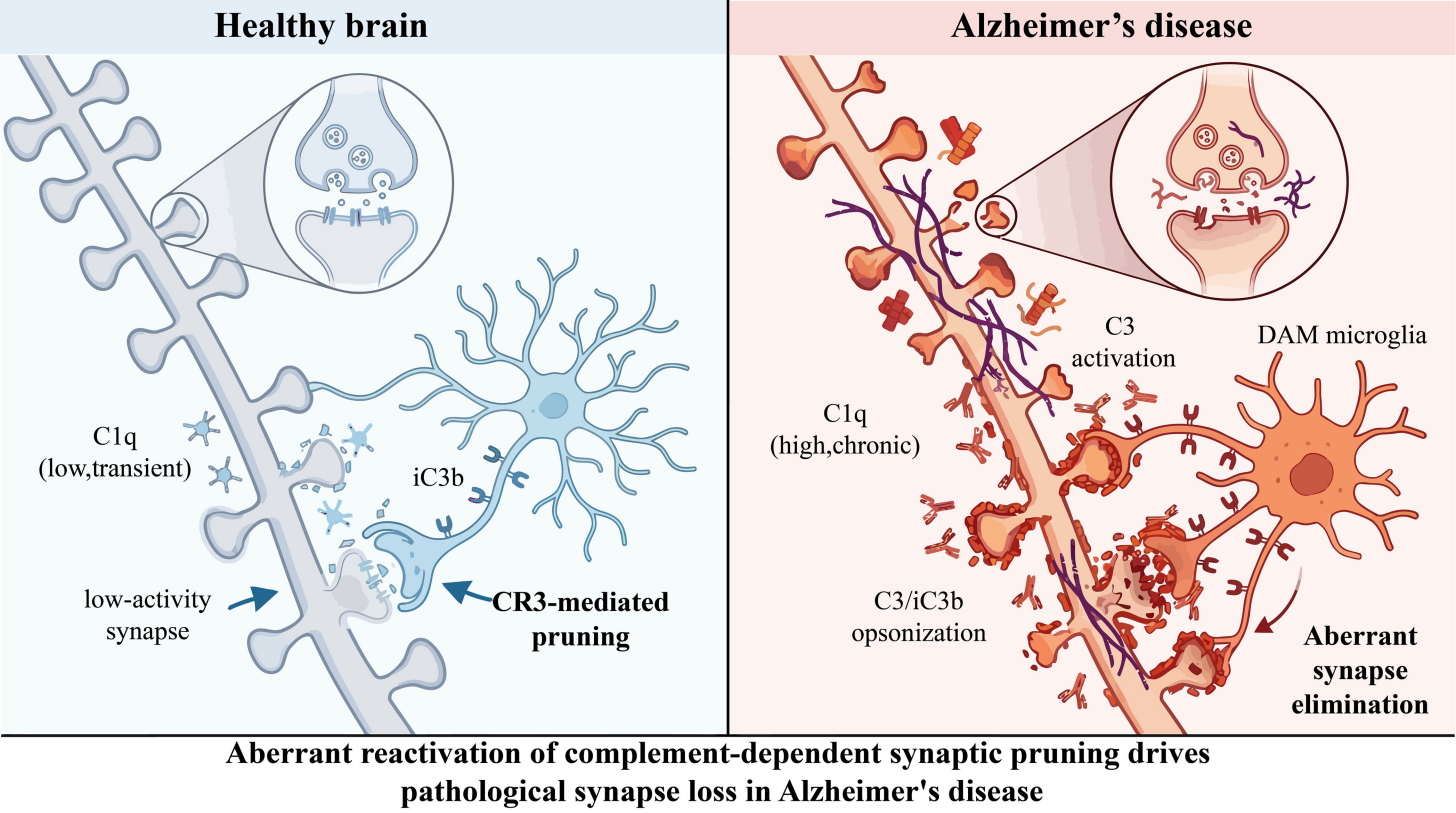
Diagram for pathological synaptic pruning of AD. This diagram illustrates complement-mediated synaptic pruning in healthy versus Alzheimer’s disease (AD) brains. In healthy brains, transient C1q marks low-activity synapses for CR3-dependent microglial pruning to maintain homeostasis. In AD, chronic C1q and persistent C3 opsonization trigger excessive DAM-mediated pruning, causing synapse loss and cognitive decline.

In Alzheimer’s disease, complement activation becomes aberrantly amplified, contributing to pathological synaptic vulnerability and chronic neuroinflammation (**Figure 2**). Disruption of the BBB may further facilitate peripheral complement infiltration, reinforcing inflammatory cascades within the CNS (54). Complement activation is not unique to AD. In Parkinson’s disease, elevated C1q and C3 deposition has been observed in the substantia nigra, where complement-mediated microglial activation may contribute to dopaminergic neuronal loss (31). In amyotrophic lateral sclerosis (ALS), increased complement components and membrane attack complex deposition have been detected in spinal motor neurons. Similar patterns of complement dysregulation have also been reported in Huntington’s disease and multiple sclerosis, supporting the notion that aberrant complement signaling represents a shared mechanism of inflammatory amplification in neurodegeneration (55).

Beyond its role as a linear proteolytic cascade, complement signaling operates as a tightly regulated amplification network characterized by multiple positive feedback loops and intrinsic inhibitory checkpoints (24). Small perturbations in activation thresholds—whether driven by genetic susceptibility, protein aggregation, or age-associated immune priming—may therefore produce disproportionately large downstream effects (56). This amplification architecture is particularly relevant in chronic neurodegenerative settings, where sustained low-grade activation can progressively shift complement function from controlled synaptic remodeling toward maladaptive inflammatory escalation. Among neurodegenerative disorders, AD provides the clearest evidence that such complement engagement evolves in a stage-dependent manner, progressively reshaping synaptic integrity, inflammatory tone, and neuronal vulnerability. This dynamic involvement forms the mechanistic basis for the complement-driven cascade discussed in the following section.

## 3 Complement-driven pathogenic cascade in Alzheimer’s disease

**Figure 3:**
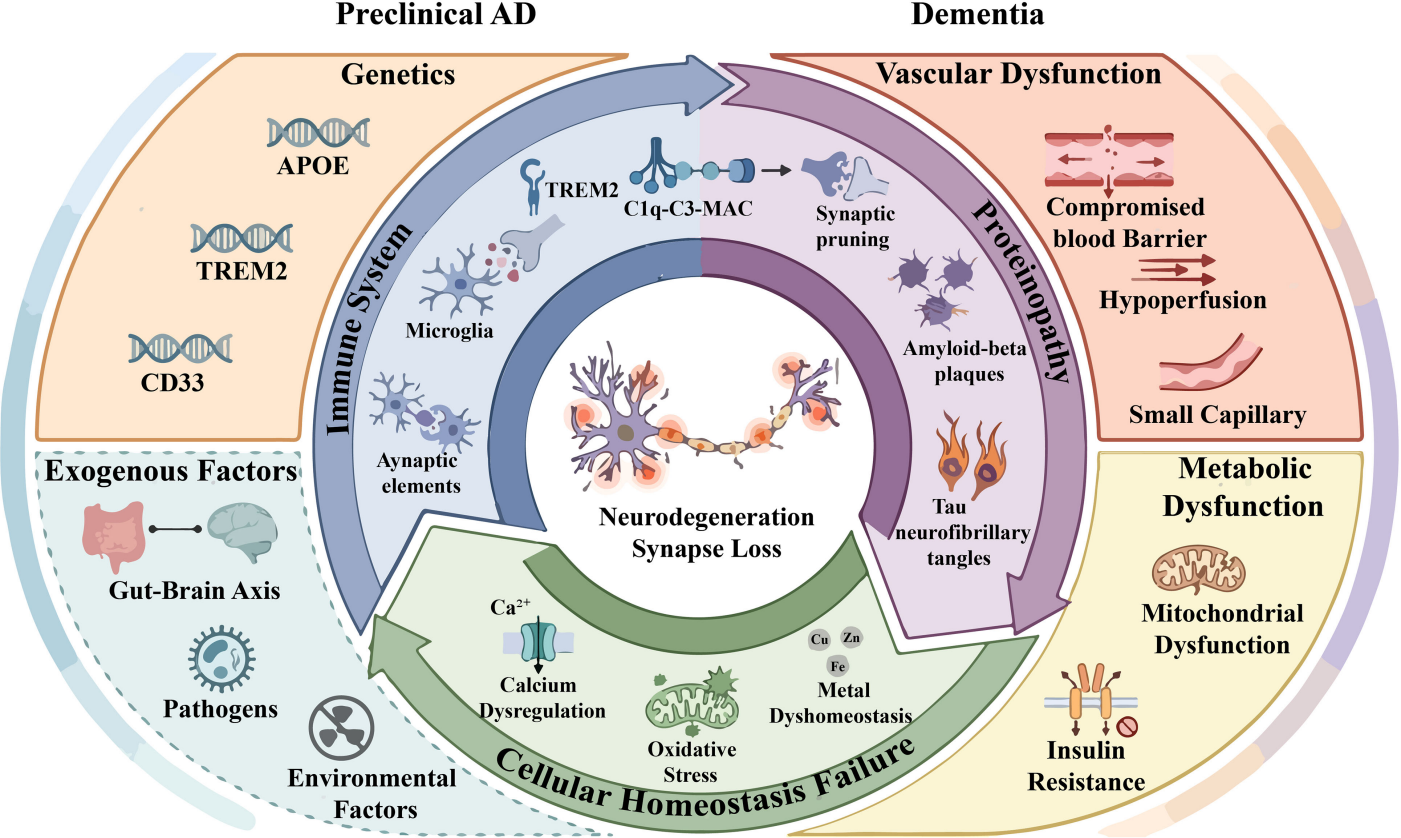
Diagram for the pathogenesis of AD. This diagram illustrates the complex pathogenic mechanisms of Alzheimer’s disease (AD), including genetics, exogenous factors, vascular dysfunction, metabolic dysfunction, cellular homeostasis failure, immune system dysfunction and proteinopathy.

As discussed in the previous section, the complement system plays a dual role in the central nervous system (CNS), functioning both as a crucial mediator of neural development and homeostasis, as well as a driver of neuroinflammation and neurodegeneration when dysregulated. In Alzheimer’s disease (AD), this duality becomes particularly evident as complement activation, initially involved in synaptic refinement, transitions into a pathological driver of disease progression (12). Recent advances in genetic, neuropathological, and experimental studies have illuminated how complement dysregulation becomes a central player in AD pathogenesis, particularly in the context of amyloid and tau pathologies (57) (**Figure 3**).

**Figure 4:**
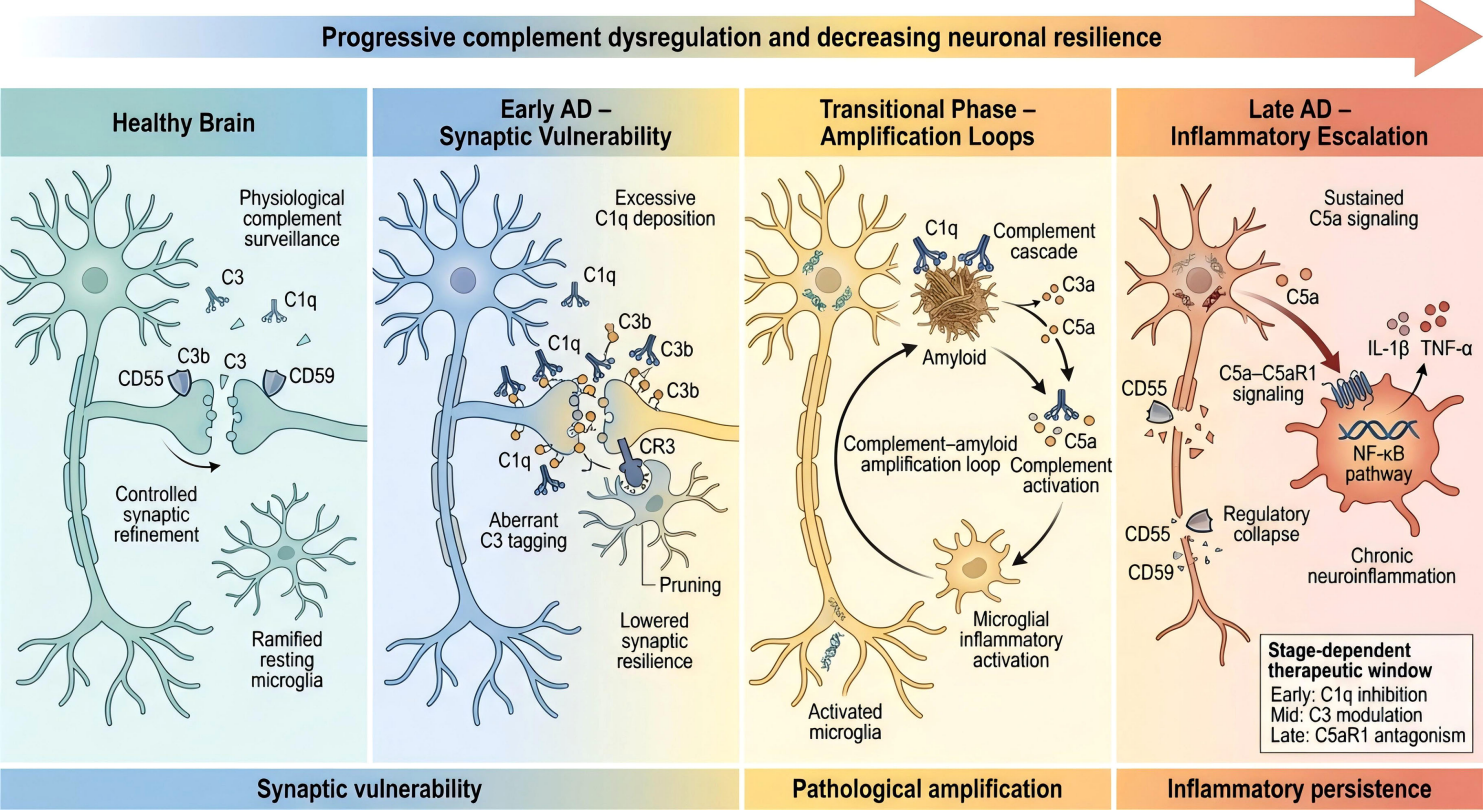
Diagram for stage-dependent model of complement dysregulation during Alzheimer’s disease progression. This schematic illustrates how complement signaling evolves from physiological synaptic surveillance to pathological neuroinflammation across Alzheimer’s disease stages. In early AD, excessive C1q and C3 tagging promotes aberrant microglial synaptic pruning. Amyloid–complement interactions then drive amplification loops and inflammatory activation. In late stages, sustained C5a–C5aR1 signaling and regulatory failure lead to chronic neuroinflammation and progressive synaptic degeneration.

In AD, complement activation follows a stage-dependent trajectory, beginning with early synaptic vulnerability and escalating to neuroinflammation and structural damage. This chapter will explore how complement signaling contributes to each of these stages, focusing on the transition from physiological functions to maladaptive immune responses that exacerbate neurodegeneration (**Figure 4**). By dissecting the evolving role of complement throughout disease progression, we aim to better understand its contribution to the pathological cascade and its potential implications for targeted intervention in the future.

### 3.1 Complement-mediated synaptic tagging and early vulnerability

Synaptic degeneration represents the anatomical feature most strongly correlated with cognitive dysfunction within the pathological milieu of AD (27). Accumulating neuropathological and experimental evidence implicates dysregulated complement signaling as a central driver of this early synaptic vulnerability, preceding overt neuronal loss and extensive plaque deposition (7).

Under physiological conditions, synaptic pruning is precisely orchestrated through coordinated interactions between microglia and complement components, particularly C1q and C3. This developmental mechanism enables the refinement of neural circuits and the removal of weak or redundant synapses (58). Mechanistically, localized calcium influx induced by synaptic weakening, reduced neuronal activity, or excitotoxic stress activates a non-apoptotic, caspase-3–dependent pathway (59). This signaling cascade promotes the externalization of phosphatidylserine residues on the synaptic membrane, generating an “eat-me” signal. Surface-exposed phosphatidylserine recruits C1q, thereby spatially restricting complement activation to specific synaptic compartments and facilitating subsequent cleavage of C3 and deposition of C3b (60). Microglia recognize these complement-opsonized synapses via complement receptor 3 (CR3) and execute targeted phagocytosis, effectively eliminating tagged connections while preserving surrounding circuitry (61).

Importantly, this process is highly activity-dependent. Synapses with reduced firing rates or impaired NMDA receptor signaling exhibit increased susceptibility to complement tagging, suggesting that complement preferentially targets functionally weakened inputs (62). Such selectivity is critical during development; however, in the aging or diseased brain, subtle perturbations in neuronal activity patterns may inappropriately lower the threshold for complement deposition. Age-associated reductions in synaptic “don’t-eat-me” signals—such as CD47—may further tip the balance toward elimination (63). Thus, physiological refinement mechanisms may become maladaptively re-engaged in vulnerable neural networks.

Astrocytes also contribute to synaptic clearance through MEGF10- and MERTK-mediated pathways and function in close coordination with microglia, particularly in AD models. Notably, these glial cell types exhibit distinct synaptic preferences: microglia preferentially eliminate inhibitory synapses, whereas astrocytes more selectively target excitatory synapses (64). This differential targeting may contribute to early excitation–inhibition imbalance observed in prodromal AD, potentially amplifying network instability before substantial neuronal death occurs.

Microglial expression of triggering receptor expressed on myeloid cells 2 (TREM2) provides an additional regulatory layer. TREM2 binds C1q with high affinity and constrains excessive complement activation at synapses, functioning as a modulatory checkpoint. In the absence of TREM2, microglia fail to transition efficiently into appropriate activation states, display impaired phagocytic regulation, and exhibit altered responses to complement-opsonized substrates (65). This dysregulation may paradoxically enhance complement-mediated synaptic loss by disrupting the fine balance between clearance and restraint.

Evidence from experimental models further supports a causal role for complement in early synaptic degeneration. Genetic deletion of C1q or C3 in amyloid mouse models preserves synaptic density and ameliorates memory deficits, despite persistent amyloid burden. Similarly, disruption of CR3 signaling attenuates microglial engulfment of synaptic elements (26). Postmortem analyses of human AD brain tissue reveal increased C1q and C3 deposition at synapses, even in early Braak stages, consistent with the view that complement engagement precedes widespread neurodegeneration (66).

Collectively, these findings indicate that complement-dependent pruning, while essential for developmental circuit refinement, becomes aberrantly reactivated in AD. At this early stage, complement signaling remains spatially confined to synaptic microdomains; however, persistent or excessive activation establishes the foundation for subsequent inflammatory amplification. The transition from selective synaptic tagging to broader immune engagement marks the first critical shift in the complement-driven pathogenic cascade.

### 3.2 Proteinopathy-associated complement amplification

#### 3.2.1 Aβ–complement axis

The emergence of pathological amyloid-β (Aβ) accumulation marks a critical transition from localized synaptic vulnerability to broader complement engagement. Multiple studies have demonstrated that Aβ species directly bind to the globular head domains of C1q, thereby initiating classical pathway activation. This interaction is not merely incidental; C1q exhibits high affinity for aggregated or misfolded protein conformations, positioning Aβ plaques as persistent activators of complement signaling within the extracellular milieu (28, 57).

Beyond classical pathway initiation, Aβ species promote robust C3 activation, which in turn fuels the alternative pathway amplification loop centered on C3bBb. This dual engagement of complement pathways establishes a feed-forward inflammatory circuit within the extracellular milieu (67). This dual engagement of classical and alternative pathways creates a robust feed-forward loop. As C3 cleavage products accumulate, anaphylatoxins such as C3a and C5a recruit and activate microglia, further increasing local complement synthesis and inflammatory signaling (21, 68).

Spatially, Aβ-driven complement activation is often concentrated around extracellular plaques. Complement components, including C1q, C3 fragments, and terminal pathway complexes, are enriched in peri-plaque regions, where activated microglia adopt pro-inflammatory phenotypes (35). In this context, complement signaling acts as an immune amplifier of amyloid pathology rather than a purely synapse-restricted tagging mechanism. The transition from synaptic refinement to plaque-associated immune activation represents a key escalation point in the complement-driven cascade.

#### 3.2.2 Tau–complement axis

Hyperphosphorylated tau pathology introduces a distinct yet convergent mode of complement activation. Similar to Aβ, pathological tau species can engage C1q and trigger complement signaling (69). However, tau-associated complement activity appears less confined to discrete extracellular deposits and more closely linked to intracellular stress, neuronal dysfunction, and inflammatory escalation.

In tauopathy models, genetic deletion of C3 significantly attenuates microglial activation and reduces synaptic degeneration without substantially altering tau burden (70). These findings indicate that complement acts downstream of tau aggregation to mediate neuronal injury, functioning as an effector rather than an initiator of tau pathology. Complement activation in this setting amplifies inflammatory cascades and promotes microglia-mediated synaptic and neuronal damage (71).

Unlike Aβ-driven complement activation, which is spatially associated with plaques, tau-driven complement engagement exhibits stronger correlation with neuronal loss and network dysfunction. As tau pathology propagates trans-synaptically across connected circuits, complement activation may spread in parallel, accompanying regions of active neurodegeneration. This pattern suggests a shift from extracellular plaque-centered complement activation to neuron-centered inflammatory amplification (49, 72).

Mechanistically, tau-induced neuronal stress may enhance complement susceptibility through altered membrane integrity, mitochondrial dysfunction, and increased exposure of phosphatidylserine or other damage-associated molecular patterns (DAMPs) (25). Complement fragments generated in this environment can further stimulate microglial metabolic reprogramming and cytokine release, creating a self-reinforcing inflammatory loop. Thus, tau pathology not only triggers complement activation but also amplifies its downstream neurotoxic consequences (73).

Collectively, these observations support the view that complement activation serves as a convergent amplifier of both amyloid and tau pathology, bridging proteotoxic stress with immune-mediated neurotoxicity. Whereas Aβ predominantly initiates plaque-associated complement activation, tau pathology appears to couple complement signaling more directly to neuronal dysfunction and loss. The coexistence of these axes establishes a multilayered amplification network in which complement activity progressively expands in spatial scope and inflammatory intensity.

This stage represents a decisive shift in the complement-driven pathogenic cascade: complement signaling transitions from localized synaptic tagging to widespread proteopathy-associated immune amplification. Persistent activation at this phase sets the stage for sustained microglial phenotypic reprogramming and broader inflammatory escalation, which are addressed in the following section.

### 3.3 Microglial phenotypic transition and inflammatory escalation

With persistent complement activation, the inflammatory landscape of the AD brain undergoes progressive transformation. What begins as spatially restricted complement engagement at synapses or around protein aggregates gradually evolves into a broader neuroimmune reprogramming process.

Sustained generation of the anaphylatoxins C3a and C5a promotes microglial recruitment and activation through engagement of C3aR and C5aR1. These potent chemoattractants not only enhance microglial motility but also trigger intracellular signaling cascades that amplify inflammatory transcriptional programs (74). Activated microglia release pro-inflammatory cytokines—including IL-1β, IL-6, IL-18, and TNF-α—as well as chemokines such as CCL1, CCL5, and CXCL1, thereby reinforcing leukocyte recruitment and local immune activation (75). Concurrently, increased production of reactive oxygen species (ROS) and nitric oxide further exacerbates oxidative stress, impairing neuronal function and synaptic integrity (76).

Importantly, complement-driven activation is accompanied by a phenotypic transition of microglia from a homeostatic surveillance state to disease-associated profiles. Transcriptomic studies have identified disease-associated microglia (DAM) and related activation states characterized by upregulation of genes involved in lipid metabolism, phagocytosis, and inflammatory signaling. Complement components—including C1q and C3—are among the genes whose expression is re-induced in these activated states, creating a self-reinforcing feed-forward loop (77). In this context, complement signaling ceases to function primarily as a synaptic tag and instead becomes embedded within a broader transcriptional amplification network.

This phenotypic shift is not merely inflammatory but also metabolic. Activated microglia undergo metabolic reprogramming, favoring glycolytic pathways over oxidative phosphorylation, a transition associated with enhanced inflammatory output (78). Complement fragments may contribute to this shift by engaging signaling pathways that converge on NF-κB activation and other pro-inflammatory transcription factors (79). As metabolic stress accumulates, microglial responses may become increasingly maladaptive, promoting chronic rather than resolving inflammation.

Astrocytes also participate in this escalation phase. Complement components released by activated microglia—particularly C1q and C3 fragments—can induce reactive astrocytic phenotypes characterized by altered synaptic support and increased inflammatory mediator production. In turn, reactive astrocytes may secrete additional complement proteins, further amplifying the inflammatory milieu. This bidirectional glial crosstalk broadens the spatial domain of complement activity, extending its impact beyond initially affected synapses or plaque-adjacent regions (80).

At this stage, complement signaling permeates the neuroimmune environment rather than remaining confined to discrete microdomains. The accumulation of diffusible complement fragments and inflammatory mediators enables propagation of immune activation across interconnected neural circuits. Persistent engagement of complement receptors sustains microglial activation, lowers activation thresholds, and increases vulnerability of neighboring neurons and synapses.

Thus, the inflammatory escalation phase represents a critical transition in the complement-driven cascade. Complement activity shifts from a structurally targeted elimination mechanism to a system-level amplifier of neuroimmune dysregulation. This expanded inflammatory state sets the conditions for terminal pathway activation and structural injury, which are discussed in the subsequent section.

### 3.4 Terminal pathway activation and structural injury

At advanced stages of complement dysregulation, activation of the terminal pathway contributes directly to structural synaptic damage. Residual C5b–C9 complexes assemble into membrane attack complexes (MACs), which integrate into neuronal membranes and disrupt synaptic architecture. Experimental analyses in the App^NL-G-F mouse model demonstrate that MAC accumulation correlates positively with disease severity, exhibits synaptic enrichment, and closely tracks the extent of synaptic injury (81). These findings indicate that terminal complement activation represents a distinct and mechanistically separable phase of complement-mediated pathology, characterized by direct structural compromise rather than purely phagocytic elimination.

Beyond simple membrane insertion, MAC formation induces a cascade of secondary injury mechanisms. Sublytic MAC deposition can trigger calcium influx, mitochondrial dysfunction, and activation of intracellular stress pathways, including MAPK and NF-κB signaling. Even in the absence of immediate lysis, these perturbations destabilize cytoskeletal elements critical for dendritic spine maintenance, impair synaptic vesicle cycling, and alter postsynaptic density organization. Consequently, synapses may undergo functional silencing prior to overt structural loss (82).

Importantly, neurons exhibit limited capacity to regulate terminal complement assembly compared to peripheral cells. While complement regulatory proteins such as CD59, CD55, and CD46 are expressed in the central nervous system, their expression can be reduced under chronic inflammatory conditions. In Alzheimer’s disease and related neurodegenerative contexts, oxidative stress and amyloid-β accumulation further compromise membrane integrity, potentially lowering the threshold for MAC insertion and amplifying vulnerability to complement-mediated injury (83, 84).

Terminal pathway activation also intersects with glial responses. MAC deposition on neuronal elements can stimulate microglia and astrocytes through damage-associated molecular patterns (DAMPs) released from injured synapses. This amplifies local inflammatory signaling, creating a feed-forward loop in which complement activation, synaptic damage, and neuroinflammation reinforce one another. Thus, the terminal pathway not only inflicts direct structural harm but also sustains a pro-degenerative microenvironment (85).

Therapeutically, these observations underscore the importance of stage-specific complement modulation. While early complement components mediate synaptic tagging and microglial pruning, terminal pathway inhibition—such as C5 blockade—may specifically prevent MAC-induced membrane disruption without completely abolishing upstream immune surveillance functions. Preclinical data suggest that limiting C5 activation reduces synaptic MAC deposition and preserves dendritic spine density, highlighting the translational potential of selectively targeting terminal complement effectors (86).

Thus, complement activity evolves from a regulated synaptic tagging mechanism into a cytotoxic effector system capable of destabilizing neuronal membranes and exacerbating neurodegeneration. Terminal pathway activation represents a late, structurally destructive phase of complement dysregulation, bridging immune signaling with irreversible synaptic injury and functional decline.

### 3.5 Context-dependent modulation of complement-driven AD pathogenesis

Aging fundamentally reshapes the regulatory landscape in which complement operates, lowering the threshold for pathological activation and amplifying synaptic vulnerability. In non-human primate studies, age-associated increases in C1q levels are accompanied by reductions in synaptic CD47 expression. CD47 normally delivers a “don’t eat me” signal that restrains microglial engulfment, whereas C1q marks synapses for clearance through interactions with postsynaptic density protein 95 (PSD95) (87). The progressive imbalance between protective and pro-phagocytic cues shifts complement signaling from selective remodeling toward excessive elimination. Thus, aging does not merely increase complement abundance; it alters the decision-making threshold governing whether a synapse is preserved or removed.

Emerging evidence further indicates that C1q may exert intracellular effects in the aged brain. Microglia-derived C1q can be internalized by neurons via endocytosis, where it associates with cytoplasmic ribonucleoprotein complexes and interferes with protein translation. This unexpected intracellular activity compromises synaptic plasticity and learning–memory processes, extending complement-mediated dysfunction beyond membrane-associated tagging and phagocytosis (22). Aging therefore broadens the mechanistic spectrum of complement injury—from extracellular opsonization to intracellular translational suppression.

Genetic background critically modulates these age-dependent shifts. One central regulatory node is the TREM2–C1q axis, which calibrates the threshold for complement-driven synaptic clearance. TREM2, expressed on microglia, senses lipid and damage-associated signals and regulates phagocytic competence. Functional TREM2 signaling constrains excessive complement activation by promoting adaptive microglial responses and maintaining homeostatic clearance pathways. However, TREM2 risk variants associated with Alzheimer’s disease (AD) impair this regulatory capacity, resulting in exaggerated C1q deposition and heightened microglial reactivity. In this context, aging-related increases in C1q are no longer effectively buffered, leading to disproportionate synaptic pruning (88). The TREM2–C1q interaction thus acts as a rheostat that determines whether complement tagging remains physiological or becomes pathogenic.

A second key checkpoint lies at the intersection of lipid metabolism and classical pathway activation, centered on apolipoprotein E (APOE). APOE isoforms differentially influence complement regulation. APOE can bind C1q and modulate classical pathway activation, positioning it as a functional checkpoint that integrates lipid transport with immune signaling. The APOE4 isoform, the strongest genetic risk factor for late-onset AD, is associated with impaired lipid homeostasis, altered membrane composition, and enhanced complement activation. Lipid dysregulation may facilitate C1q binding and membrane vulnerability, thereby coupling metabolic stress to complement-mediated injury. This lipid–complement crosstalk provides a mechanistic explanation for how APOE genotype modifies disease trajectory and synaptic resilience during aging (89, 90).

At the transcriptional level, TYROBP (DAP12) functions as a microglial signaling adaptor that amplifies complement-related gene expression. As a downstream partner of TREM2 and other immunoreceptors, TYROBP coordinates a disease-associated microglial (DAM) transcriptional program characterized by upregulation of complement components, phagocytic receptors, and inflammatory mediators (91). In aged or genetically susceptible brains, TYROBP-centered reprogramming acts as a transcriptional amplifier, reinforcing complement activation loops (92). Rather than initiating pathology independently, this amplification magnifies pre-existing complement signals, accelerating the transition from adaptive immune surveillance to chronic neuroinflammation.

Importantly, neurons themselves retain intrinsic mechanisms that restrain complement-mediated damage. Neuronal pentraxin 2 (Nptx2) has emerged as a local inhibitory regulator that stabilizes excitatory synapses and counterbalances complement tagging. Nptx2 supports synaptic integrity and may indirectly limit C1q deposition by maintaining functional synaptic activity. Declines in Nptx2 expression observed in aging and early AD weaken this neuronal “brake,” rendering synapses more susceptible to complement recognition and elimination. Loss of Nptx2 therefore represents a failure of local resistance that synergizes with microglial and systemic changes (93).

In parallel, elevated levels of granzyme K (GZMK) in aged central and peripheral immune compartments may further potentiate complement activation and inflammatory signaling, creating a permissive environment for synaptic vulnerability (94). Together, these findings illustrate that complement-driven pathology is not determined solely by complement abundance but by a multilayered regulatory network shaped by aging, lipid metabolism, transcriptional state, and neuronal resilience.

Collectively, aging and genetic risk factors converge to recalibrate complement activity at multiple levels: the synaptic tagging threshold (TREM2–C1q), classical pathway initiation (APOE), microglial transcriptional amplification (TYROBP), and neuronal intrinsic restraint (Nptx2). This integrated framework helps explain model-dependent variability and stage-specific outcomes in experimental studies, positioning complement not as an isolated effector cascade but as a context-sensitive system whose pathogenic potential emerges from age- and genotype-dependent regulatory imbalance.

### 3.6 Dysregulation of complement regulatory systems in AD

Complement activation in the healthy brain is tightly controlled by membrane-bound and soluble regulatory proteins that restrict excessive cascade amplification. In the context of AD, emerging genetic and transcriptomic evidence suggests that alterations in complement regulatory pathways may contribute to disease susceptibility, although direct functional validation in AD models remains limited (95).

Genome-wide association studies have identified complement receptor 1 (CR1) as a risk locus for AD, implicating impaired C3b clearance or altered complement complex handling in disease pathogenesis. Similarly, clusterin (CLU), a known inhibitor of terminal complement complex assembly, has been genetically associated with AD, raising the possibility that dysregulation of terminal pathway control may influence disease progression (96).

Transcriptomic analyses of AD brain tissue and single-cell sequencing datasets have reported altered expression patterns of several complement-related regulators, including complement factor H (CFH), CD55, and CD59, although these findings vary across cohorts and disease stages. Given the established role of CD55 and CD59 in limiting convertase stability and preventing membrane attack complex formation, even subtle reductions in their activity could theoretically lower the threshold for terminal pathway activation (97).

Taken together, current evidence supports the hypothesis that complement dysregulation in AD may reflect not only excessive activation but also insufficient regulatory restraint. However, systematic experimental studies specifically interrogating complement regulatory proteins in AD models are still needed to define their causal contribution.

### 3.7 Conceptual integration: a stage-dependent complement cascade model

Taken together, the evidence presented across Sections 3.1–3.6 supports a unified model in which complement activation in Alzheimer’s disease follows a stage-dependent trajectory characterized by shifting functional hierarchies. In early phases, localized C1q-mediated synaptic tagging operates within discrete microdomains, selectively targeting vulnerable synapses. As amyloid and tau pathologies accumulate, complement engagement expands beyond synaptic compartments and becomes embedded within proteopathy-associated amplification loops. Sustained activation subsequently drives microglial phenotypic reprogramming and inflammatory escalation, culminating in terminal pathway activation and structural synaptic injury.

Importantly, this cascade is not governed solely by complement abundance but by context-dependent regulatory thresholds shaped by aging, genetic background, lipid metabolism, and neuronal resilience mechanisms. Dysregulation emerges when physiological refinement processes exceed homeostatic control, transforming complement signaling from a circuit-modulating system into a self-reinforcing neurodegenerative amplifier.

This stage-dependent complement cascade framework provides a mechanistic foundation for understanding the temporal heterogeneity of AD progression and offers a conceptual bridge to translational considerations, including biomarker development, therapeutic timing, and patient stratification strategies discussed in the following section.

## 4 Translational implications of complement dysregulation in AD

The stage-dependent complement cascade model outlined above underscores the dynamic and context-sensitive nature of complement engagement throughout Alzheimer’s disease progression. If complement dysregulation indeed functions as an early synaptic vulnerability driver and later inflammatory amplifier, then its clinical implications extend beyond mechanistic curiosity. A central question therefore emerges: can complement biology be translated into meaningful diagnostic and therapeutic strategies?

Despite compelling experimental evidence positioning complement as a core pathogenic axis, its integration into clinical practice remains limited. This gap between mechanistic insight and translational application necessitates careful evaluation of both the opportunities and structural constraints associated with complement-based interventions.

### 4.1 Clinical promise and translational gap

The current clinical diagnosis of Alzheimer’s disease (AD) relies predominantly on cerebrospinal fluid (CSF) biomarkers and neuroimaging modalities (98). Although these approaches provide robust diagnostic accuracy, their implementation is constrained by high cost, limited accessibility, and the invasive nature of lumbar puncture (99). Consequently, there has been growing interest in minimally invasive, blood-based biomarkers capable of supporting large-scale screening and longitudinal monitoring (100).

Genetic and biomarker studies converge in implicating complement dysregulation in AD pathogenesis. Genome-wide association studies (GWAS) have identified complement-related loci, including CR1 and CLU, as risk factors for AD, suggesting that complement alterations may precede or accompany disease onset. Among circulating complement components, C1q has emerged as one of the most extensively investigated candidates. A multi-parameter plasma model incorporating clusterin, complement factor I (CFI), and the terminal complement complex (TCC) achieved a predictive accuracy of 85% for progression from mild cognitive impairment (MCI) to AD (101). More recently, a cohort study involving 574 participants demonstrated significantly elevated plasma C1q levels in AD patients compared with cognitively normal controls (AUC = 0.79; sensitivity 76%; specificity 72%), outperforming several other complement proteins (102). Notably, this elevation appeared independent of APOE ϵ4 genotype, indicating that complement markers may provide information beyond established genetic risk factors.

Beyond cross-sectional discrimination, circulating C1q levels have been reported to correlate positively with CSF pTau/tTau ratios and to remain elevated during clinical progression from MCI to AD. These findings suggest that complement activation may reflect ongoing neuroinflammatory burden and neuronal injury, supporting its potential role as a dynamic biomarker for disease staging and progression monitoring (103).

Despite these encouraging observations, complement-based biomarkers have not yet achieved clinical standardization. Disease specificity remains limited, as elevated C1q levels are also observed in systemic inflammatory and autoimmune conditions (104). In addition, large-scale population validation comparable to that performed for established CSF biomarkers is still lacking. Thus, complement components are more plausibly positioned as contributors to multi-marker panels rather than standalone diagnostic tools.

A similar pattern emerges in the therapeutic domain. Extensive preclinical studies demonstrate that genetic deletion or pharmacological inhibition of complement components can rescue synapse loss and improve cognitive performance in AD models (7, 8, 69). However, no complement-targeted therapy has yet advanced through or completed phase I clinical evaluation specifically for AD. This striking discrepancy between mechanistic robustness and clinical stagnation suggests the presence of structural translational barriers rather than a simple delay in therapeutic development.

### 4.2 Structural barriers and unresolved controversies

The limited clinical translation of complement-based strategies reflects several interconnected biological and practical challenges.

First, complement signaling in the central nervous system exhibits pronounced context dependence. Experimental evidence indicates that complement components may exert protective roles during early development and possibly in initial stages of pathology, while becoming maladaptive when activation is excessive or sustained (105). This stage-dependent duality complicates therapeutic targeting. Indiscriminate or prolonged inhibition may interfere with physiological synaptic remodeling or beneficial immune surveillance, whereas delayed intervention may fail to prevent irreversible synaptic injury (106). Thus, one unresolved controversy concerns whether complement activation functions primarily as an upstream disease driver or as a context-dependent amplifier of ongoing pathology. Clarifying this distinction is essential for rational target selection and timing of intervention.

Second, complement represents a core effector system of innate immunity. Systemic blockade—particularly at central nodes such as C3—carries potential risks, including increased susceptibility to infection and impaired host defense (107). These safety concerns impose inherent constraints on dosing intensity, duration of treatment, and patient selection. Therapeutic strategies must therefore balance sufficient modulation of pathological complement activity within the brain against preservation of systemic immune competence.

Third, drug delivery remains a significant technical barrier. Most complement-directed biologics are administered systemically and demonstrate limited penetration across the blood–brain barrier (BBB), often below 1% of circulating levels (108). Although emerging approaches—including receptor-mediated transcytosis platforms and focused ultrasound combined with microbubbles—have shown promise in enhancing central nervous system exposure, these technologies introduce additional complexity, cost, and safety considerations (109). The feasibility of sustained, long-term modulation of complement activity within the brain remains uncertain.

Finally, trial design misalignment may have contributed to the slow clinical progress in this field. Complement activation evolves dynamically across disease stages, yet most therapeutic paradigms in AD have not incorporated stage-specific enrollment criteria or biomarker-guided stratification. Endpoints commonly used in amyloid-targeting trials may not adequately capture synaptic preservation or inflammatory modulation, which represent primary mechanistic outputs of complement intervention. Without alignment between biological mechanism and clinical outcome measures, therapeutic effects may remain undetected even if mechanistically meaningful (110, 111).

Taken together, these biological ambiguities, safety constraints, delivery challenges, and design limitations help explain why complement-targeted strategies—despite strong mechanistic rationale—have not yet translated into established clinical interventions. Addressing these structural barriers will be essential for redefining the therapeutic role of complement modulation within the broader AD treatment landscape.

### 4.3 Complement-based therapy in the AD treatment Landscape

The therapeutic landscape of Alzheimer’s disease has historically been dominated by strategies targeting amyloid-β and, more recently, tau pathology. Although these proteopathy-centered approaches have demonstrated the capacity to reduce pathological burden, their clinical benefits remain modest and stage-dependent (3, 4). In this context, complement modulation occupies a distinct and potentially integrative position within the broader treatment framework.

Rather than directly targeting protein aggregation, complement-based strategies aim to recalibrate the neuroimmune environment that links proteotoxic stress to synaptic and neuronal injury. In this sense, complement signaling represents a mechanistic bridge between pathological protein deposition and immune-mediated synaptic degeneration (112). Therapeutically, complement modulation is therefore best conceptualized not as a replacement for proteopathy-directed therapies, but as an immunological intervention that addresses a parallel and interacting pathogenic axis.

Importantly, the therapeutic role of complement is unlikely to be uniform across disease stages. Based on the stage-dependent cascade model discussed in this review, early complement activity—particularly C1q-mediated synaptic tagging—may represent a window for disease-modifying intervention. In preclinical models, inhibition of early complement components preserves synaptic density and cognitive performance even in the presence of persistent amyloid burden, suggesting that synaptic protection may be partially dissociable from plaque clearance (113). In this early phase, complement modulation could theoretically function as a strategy to maintain synaptic resilience and delay the transition from preclinical pathology to symptomatic impairment.

By contrast, during intermediate or established stages of AD, complement activation appears increasingly embedded within broader inflammatory amplification networks. At this stage, selective targeting of downstream effectors—such as the C5a–C5aR1 axis—may serve primarily to dampen inflammatory escalation rather than to reverse core pathology (114). In late stages characterized by structural injury and terminal pathway activation, complement inhibition is more plausibly positioned as a neuroprotective or disease-slowing adjunct rather than a fundamentally disease-modifying therapy.

These considerations underscore that complement-targeted intervention should be viewed as a stage-informed immunomodulatory strategy. It is unlikely to function as a universal monotherapy capable of independently halting AD progression (26). Instead, complement modulation may exert maximal benefit when deployed in combination with proteopathy-directed agents, particularly in biomarker-selected subgroups exhibiting evidence of complement overactivation (115). In such a framework, amyloid- or tau-targeting therapies could reduce upstream proteotoxic triggers, while complement modulation preserves synaptic integrity and mitigates inflammatory amplification.

Another critical positioning consideration concerns patient heterogeneity. Complement activation levels vary across individuals and may be influenced by genetic background, age, and inflammatory comorbidities (116). This variability suggests that complement-based therapies may be most effective in biologically defined subpopulations—such as individuals with elevated plasma or CSF complement markers, or those carrying complement-related genetic risk variants—rather than in unselected AD cohorts (101).

Taken together, complement-based therapy should not be conceptualized as a standalone anti-amyloid alternative, nor as a nonspecific anti-inflammatory approach. Rather, it represents a precision immunological strategy aimed at restoring balance within a dysregulated complement–synapse–microglia axis. Its therapeutic potential lies in stage-specific deployment, combination paradigms, and biomarker-guided patient selection. Clarifying these parameters will be essential for determining whether complement modulation can meaningfully alter the trajectory of AD in clinical practice.

### 4.4 Strategic priorities and framework for future clinical development

The limited clinical translation of complement-targeted strategies in AD does not reflect a lack of mechanistic rationale, but rather the absence of a coherent, stage-informed development strategy. To advance the field, future efforts must move beyond broad inhibition paradigms and instead prioritize mechanistically grounded, stratified approaches. Based on the stage-dependent cascade model outlined in this review, several strategic priorities emerge.

#### 4.4.1 Priority mechanistic hypotheses for validation

First, the C1q-mediated synaptic tagging axis warrants priority validation as an early driver of vulnerability. Accumulating experimental evidence suggests that aberrant reactivation of developmental pruning mechanisms precedes widespread neuronal loss (7). However, definitive human validation remains incomplete. Longitudinal studies integrating plasma or CSF C1q measurements with synaptic imaging markers—such as SV2A PET—could clarify whether early complement deposition predicts subsequent synaptic decline (101). Establishing temporal causality in humans is essential before large-scale early-intervention trials are undertaken.

Second, the C5a–C5aR1 axis should be evaluated as a principal inflammatory amplifier in intermediate stages of disease. Unlike upstream complement components that participate in synaptic tagging, C5a signaling predominantly drives microglial chemotaxis and inflammatory gene expression. Targeting this downstream effector may therefore attenuate inflammatory escalation while preserving upstream immune surveillance functions. Selective modulation at this level may offer a more favorable safety profile compared with central complement blockade. By contrast, global inhibition of C3, despite its central hub position in the cascade, presents substantial systemic risk and may prove excessively disruptive. Thus, broad upstream blockade should be approached cautiously unless delivery systems and safety monitoring strategies can be substantially refined (29, 117).

#### 4.4.2 Stage-specific therapeutic windows

Complement modulation should be aligned with disease stage. In preclinical or prodromal phases characterized by biomarker positivity but preserved cognition, early intervention aimed at limiting C1q-dependent synaptic vulnerability may have the greatest disease-modifying potential. In mild-to-moderate stages, strategies targeting inflammatory amplification (e.g., C5aR1 antagonism) may function to stabilize disease progression. In advanced disease, terminal pathway modulation may primarily serve neuroprotective or symptom-stabilizing roles rather than reversing pathology.

This stage-specific framework implies that complement-based therapies should not be evaluated under a uniform treatment paradigm. Instead, distinct agents may be optimized for distinct phases of disease, analogous to oncology models in which therapeutic selection is tailored to tumor stage and molecular profile (118).

#### 4.4.3 Biomarker-guided patient stratification

A central obstacle in prior immunomodulatory trials has been the biological heterogeneity of enrolled cohorts. Complement activation is unlikely to be uniformly elevated across all individuals with Alzheimer’s disease, and indiscriminate enrollment may dilute therapeutic signals. Therefore, future trials should incorporate complement-informed stratification strategies designed to enrich for biologically relevant subgroups (119).

One pragmatic approach involves selecting participants with evidence of heightened complement activation, as indicated by elevated plasma or cerebrospinal fluid levels of C1q, C3 fragments, or terminal pathway activation products. Such enrichment would align therapeutic targeting with underlying pathophysiology rather than relying solely on clinical diagnosis. In parallel, incorporation of complement-related genetic variants—such as CR1 or CLU—into risk stratification frameworks may further refine patient selection by identifying individuals with intrinsic susceptibility to complement dysregulation (120).

Importantly, complement markers should not be considered in isolation. Their integration with established AD biomarkers—including Aβ42 and phosphorylated tau—may enable the identification of biologically coherent subgroups characterized by concurrent proteopathic burden and immune amplification. This multimodal stratification paradigm could increase the probability of detecting meaningful therapeutic effects while reducing heterogeneity-driven variability that has limited prior anti-inflammatory trials.

#### 4.4.4 Mechanism-aligned endpoints

Beyond patient selection, endpoint design must be explicitly aligned with the biological mechanism of complement-targeted intervention. A recurring limitation of previous AD trials has been the reliance on outcome measures that do not adequately capture the primary mechanistic targets of the therapy under investigation. If complement modulation is intended to preserve synaptic integrity and attenuate inflammatory amplification, then endpoints should extend beyond reductions in amyloid plaque burden (121).

Synaptic density imaging, such as SV2A PET, offers a direct measure of structural synaptic preservation and may provide a more sensitive readout of complement-mediated synaptic protection (122). In parallel, dynamic assessment of complement activation fragments—including C3a, C5a, and soluble C5b-9—in cerebrospinal fluid could serve as pharmacodynamic markers of pathway engagement. Broader neuroinflammatory signatures in fluid biomarkers may further clarify whether immune recalibration is achieved (123).

Taken together, a stage-informed, biomarker-guided, and mechanism-aligned development strategy offers a coherent path forward for complement-based intervention in AD. Rather than pursuing indiscriminate inhibition of the complement cascade, future efforts should prioritize selective modulation tailored to disease phase and biological context. Only through such precision-oriented frameworks can the translational potential of complement modulation be rigorously evaluated and, if validated, integrated into the evolving therapeutic landscape of Alzheimer’s disease.

## 5 Conclusion

Alzheimer’s disease has long been conceptualized primarily through the lens of amyloid and tau pathology. While these proteinopathies remain central to disease biology, mounting evidence indicates that they do not act in isolation. Rather, they operate within a broader network of immune, metabolic, vascular, and genetic interactions that collectively shape disease trajectory. Within this multifactorial landscape, complement signaling emerges not as a secondary bystander response, but as a dynamic and stage-sensitive regulatory axis that links proteotoxic stress to synaptic vulnerability and neuroinflammatory escalation.

This review has outlined a stage-dependent complement cascade model in which complement activity evolves from localized synaptic tagging to widespread inflammatory amplification and, ultimately, structural injury. In early disease phases, C1q-mediated synaptic pruning mechanisms—physiological during development—appear to be aberrantly reactivated, lowering the threshold for synaptic elimination. As amyloid and tau pathology accumulate, complement engagement expands into proteopathy-associated amplification loops that drive microglial phenotypic reprogramming and sustained inflammatory signaling. In later stages, terminal pathway activation contributes directly to structural membrane damage and synaptic destabilization. Importantly, this progression is not determined solely by complement abundance, but by context-dependent regulatory imbalance shaped by aging, genetic background, lipid metabolism, and neuronal resilience mechanisms.

Despite compelling mechanistic evidence, complement-targeted strategies have not yet translated into established clinical interventions. This translational gap reflects biological duality, systemic safety constraints, delivery challenges, and historical misalignment between therapeutic mechanism and trial design. Complement modulation is unlikely to function as a universal anti-amyloid alternative or a nonspecific anti-inflammatory approach. Instead, its therapeutic value may lie in precision-oriented, stage-informed deployment—potentially as an adjunct to proteopathy-directed therapies in biomarker-defined subgroups exhibiting complement overactivation.

Future progress will depend on prioritizing specific mechanistic hypotheses, particularly those centered on early C1q-mediated synaptic vulnerability and intermediate C5a-driven inflammatory amplification. Equally critical will be the integration of complement-informed patient stratification and mechanism-aligned endpoints capable of capturing synaptic preservation and immune recalibration. Without such conceptual and methodological alignment, biologically meaningful effects may remain obscured.

More broadly, the study of complement in Alzheimer’s disease challenges traditional linear models of neurodegeneration. It highlights how innate immune signaling, when operating beyond regulatory thresholds, can transform physiological circuit refinement into pathological synaptic loss. Clarifying when, where, and in whom complement activity shifts from adaptive to maladaptive will be central to determining its clinical utility. If rigorously evaluated within stage-specific and biologically stratified frameworks, complement modulation may contribute to a more nuanced and multidimensional therapeutic paradigm—one that acknowledges Alzheimer’s disease not as a single-cause disorder, but as a dynamically evolving network pathology.
